# Supplementary material for: Therapist-Guided Versus Self-Guided Forest Immersion: Comparative Efficacy on Short-Term Mental Health and Economic Value
Source: Behav Sci (Basel). 2025 Nov 24;15(12):1618. doi: 10.3390/bs15121618 (PMC12729753; doi:10.3390/bs15121618)
Supplement: Supplementary file 1 [file behavsci-15-01618-s001.zip › behavsci-3941826-supplementary.pdf]

Article

Therapist-Guided Versus Self-Guided Forest Immersion: Comparative Efficacy on Short-Term Mental Health and Economic Value

Rosa Rivieccio [1,†], Francesco Meneguzzo [2,3,*,†], Giovanni Margheritini [3], Tania Re [4], Ubaldo Riccucci [5] and Federica Zabini [2]

[1] Centro di Ricerca Politiche e Bioeconomia, Consiglio per la Ricerca in Agricoltura e l'Analisi dell'Economia Agraria, I-00178 Roma, Italy; rosa.rivieccio@crea.gov.it
[2] Institute of Bioeconomy, National Research Council, 10 Via Madonna del Piano, I-50019 Sesto Fiorentino, Italy; federica.zabini@cnr.it
[3] Central Scientific Committee, Italian Alpine Club, I-20124 Milano, Italy; margheritinig@gmail.com
[4] Group Anthropology of Health—Biosphere and Healing Systems, University of Genoa, I-16128 Genoa, Italy; tania.re77@gmail.com
[5] Simple Departmental Operating Unit Anesthesia and Resuscitation, Cecina Hospital, I-57023 Cecina, Italy; ubaldo.riccucci@uslnordovest.toscana.it
* Correspondence: francesco.meneguzzo@cnr.it; Tel.: +39-392-9850002
† These authors contributed equally to this work.

Supplementary materials

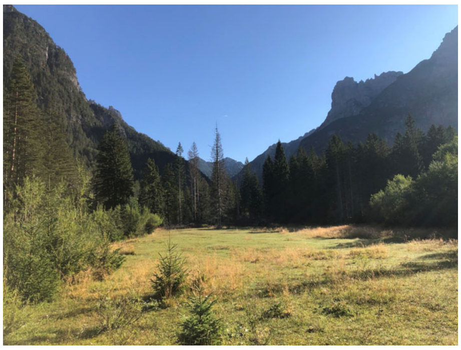

(a)

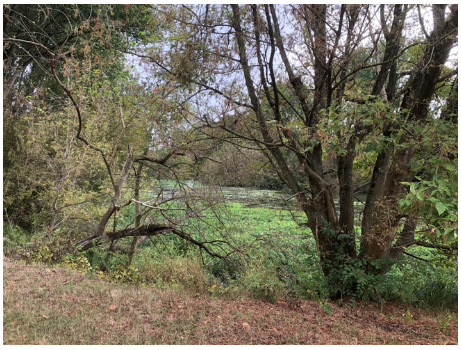

(b)

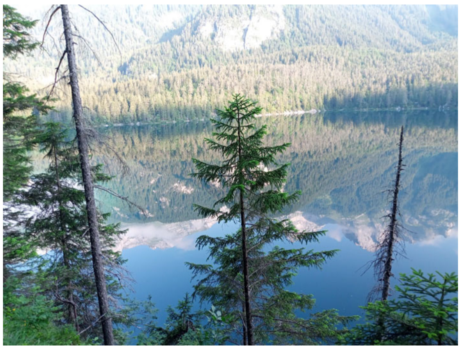

(c)

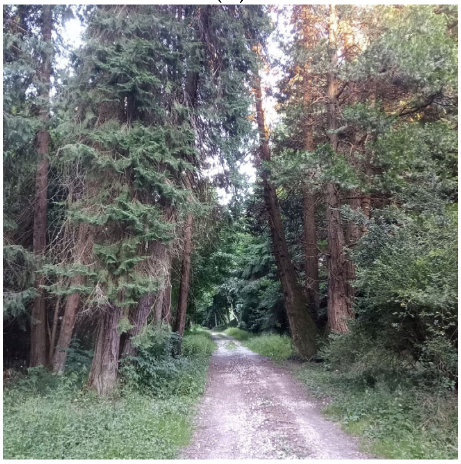

(d)

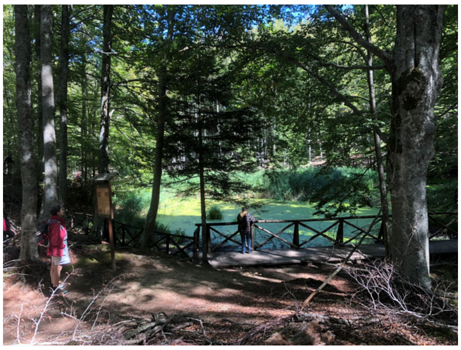

(e)

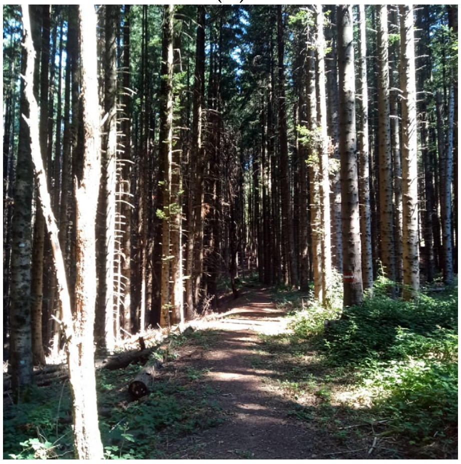

(f)

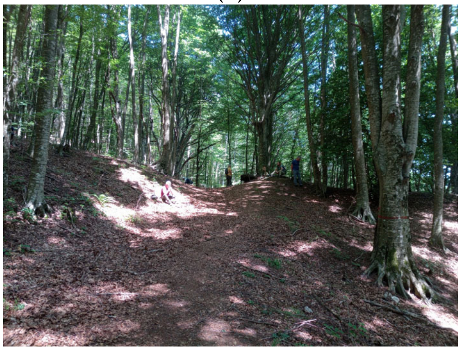

(g)

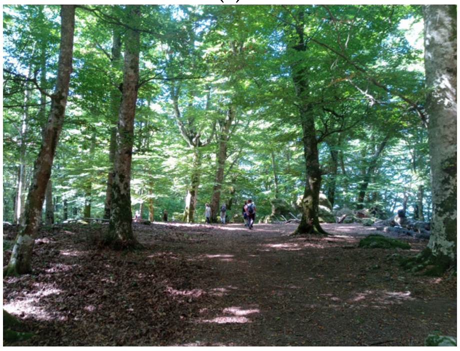

(h)

Figure S1. Representative pictures of the local environments. (a) S1; (b) S2; (c) S3; (d) S4; (e) S5; (f) S6; (g) S7; (h) S8. Photos: F. Meneguzzo.

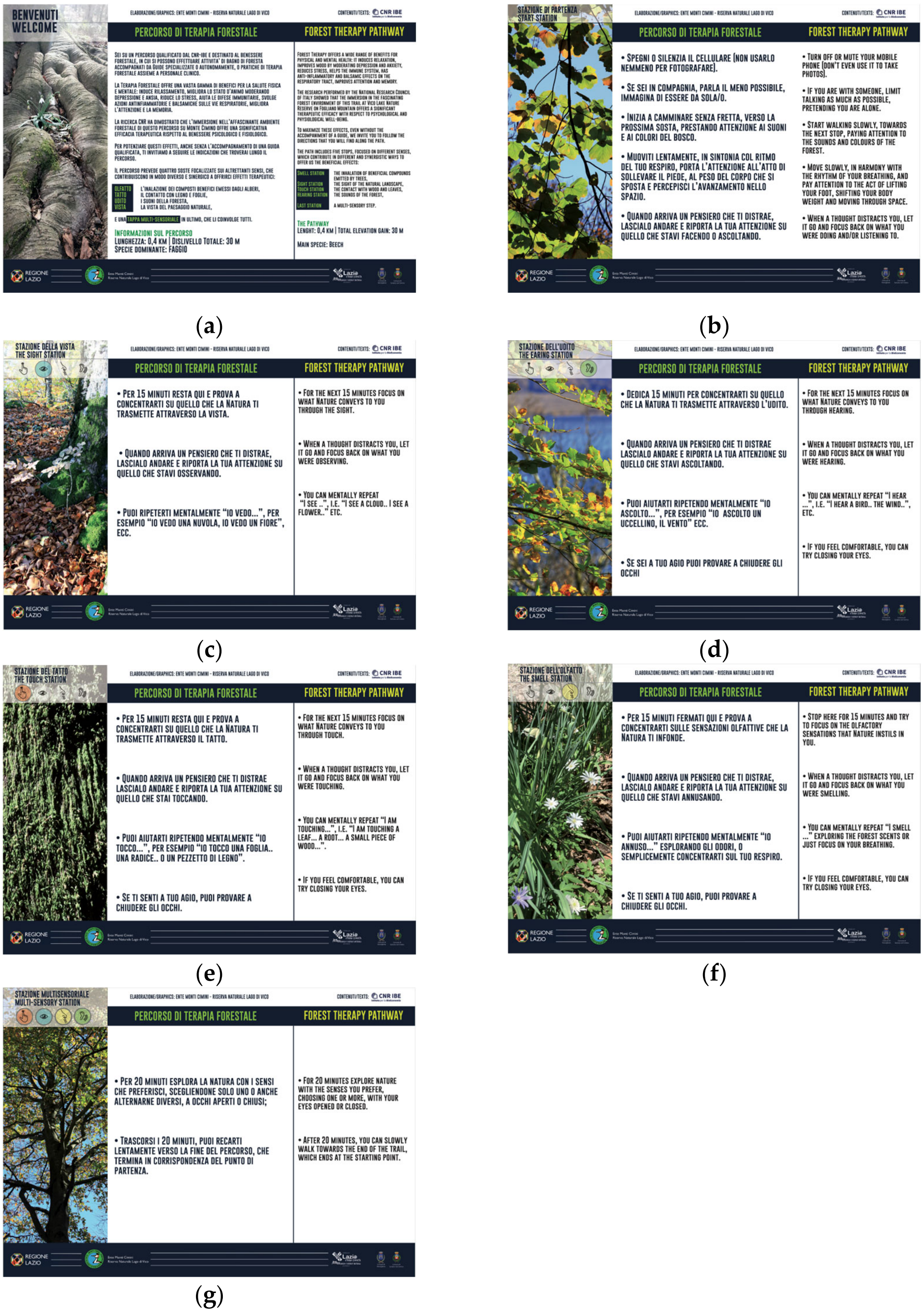

Figure S2. Sample boards with the instructions read by the participants (SG sessions) or verbally communicated by the therapist (TG sessions), in the Italian and English languages. (**a**) Presentation of the forest therapy trail; (**b**) General instructions to participants; (c) Instructions for a stop focused on the sense of sight; (**d**) Instructions for a stop focused on the sense of hearing; (**e**) Instructions for a stop focused on the sense of touch; (**f**) Instructions for a stop focused on the sense of smelling; (**g**) Instructions for a stop focused on the multiple senses. Graphics: A. Sasso, Cimini Mountains Authority - Lake Vico Regional Nature Reserve, reproduced with kind permission of the author.

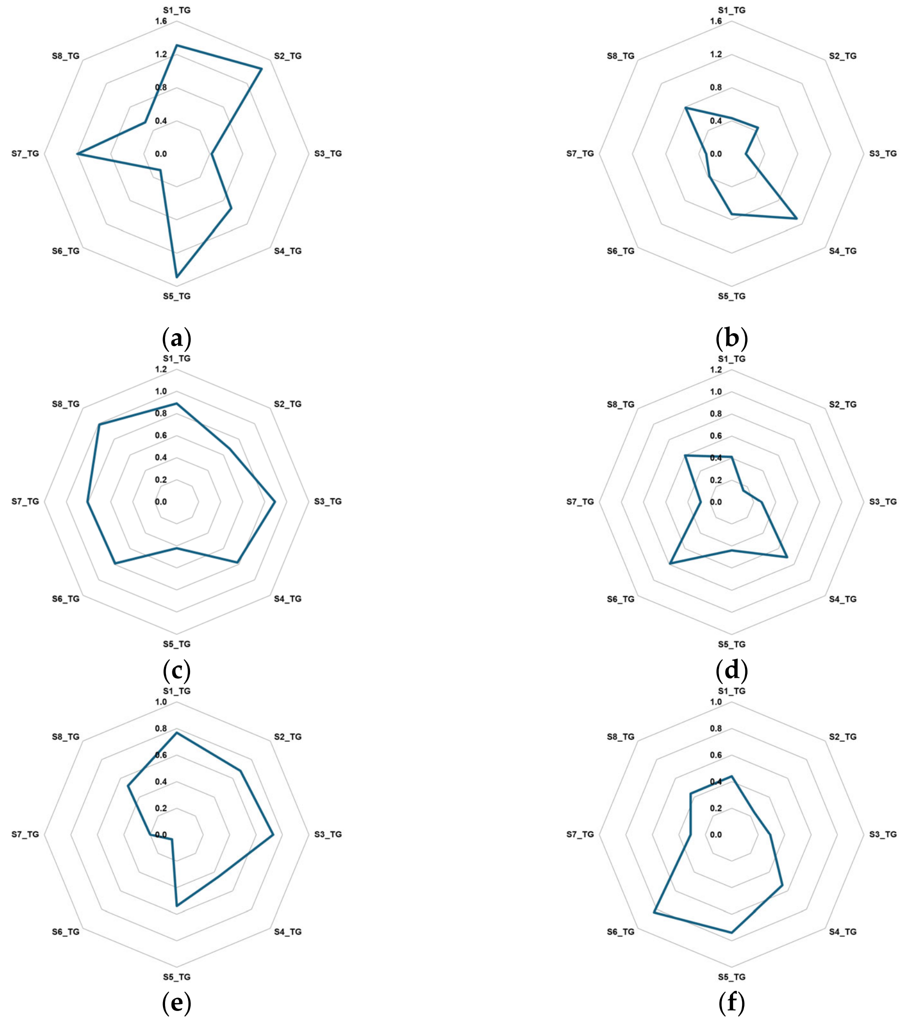

Figure S3. Radar-plot style charts using the Cohen's d values from Table 4. (a) TG sessions, STAI-S; (b) SG sessions, STAI-S; (c) TG sessions, POMS-esteem; (d) SG sessions, POMS-esteem; (e) TG sessions, POMS-TMD; (f) SG sessions, POMS-TMD.

Table S1. Estimate of annual economic value of TG and SG sessions for the lower threshold of 20,000 EUR/QALY, with progressive inclusion of STAI-S, POMS-TMD and POMS-esteem.

ID	€ per person, annual		
	STAI-S	STAI-S + POMS-TMD	STAI-S + POMS-TMD + POMS-esteem
S1_TG	3261 (95% C.I. 2094 to 5042)	2596 (95% C.I. 1698 to 4142)	4967 (95% C.I. 2832 to 8839)
S1_SG	1634 (95% C.I. 264 to 3272)	1537 (95% C.I. 501 to 3342)	1537 (95% C.I. 687 to 5118)
S2_TG	2394 (95% C.I. 1339 to 4040)	2394 (95% C.I. 1435 to 5363)	5313 (95% C.I. 2654 to 11,469)
S2_SG	1167 (95% C.I. 167 to 2927)	1167 (95% C.I. 266 to 4121)	1167 (95% C.I. 332 to 4632)
S3_TG	1183 (95% C.I. 348 to 2278)	2198 (95% C.I. 1216 to 3406)	5438 (95% C.I. 2796 to 9097)
S3_SG	0 (95% C.I. 0 to 2169)	0 (95% C.I. 0 to 5818)	0 (95% C.I. 0 to 10,396)
S4_TG	4026 (95% C.I. 1167 to 7799)	4026 (95% C.I. 1592 to 7472)	3765 (95% C.I. 2018 to 8017)
S4_SG	5221 (95% C.I. 2578 to 10,254)	5221 (95% C.I. 1901 to 8945)	5221 (95% C.I. 2628 to 9103)
S5_TG	2914 (95% C.I. 2135 to 4391)	2617 (95% C.I. 1966 to 5588)	2617 (95% C.I. 2066 to 6564)
S5_SG	2481 (95% C.I. 1264 to 5370)	2845 (95% C.I. 1746 to 5921)	4588 (95% C.I. 2499 to 9857)
S6_TG	0 (95% C.I. 0 to 2071)	0 (95% C.I. 0 to 5341)	2909 (95% C.I. 984 to 9155)
S6_SG	0 (95% C.I. 0 to 2496)	0 (95% C.I. 1771 to 2823)	3386 (95% C.I. 1804 to 10,654)
S7_TG	2057 (95% C.I. 811 to 5595)	2057 (95% C.I. 809 to 5472)	3678 (95% C.I. 2292 to 7616)
S7_SG	0 (95% C.I. 0 to 4605)	0 (95% C.I. 0 to 4605)	0 (95% C.I. 0 to 4605)
S8_TG	1766 (95% C.I. 947 to 3147)	1944 (95% C.I. 1136 to 3339)	4386 (95% C.I. 2632 to 7119)
S8_SG	2812 (95% C.I. 1528 to 4823)	2812 (95% C.I. 1523 to 4706)	3802 (95% C.I. 2439 to 6305)
